# Folate intake and the risk of endometrial cancer: A dose–response meta-analysis

**DOI:** 10.1097/MD.0000000000039775

**Published:** 2024-09-20

**Authors:** Jiaye Long, Du Wang, Miyang Yang, Yingrong Pang, Meiqiong Li, Shuxin Qin, Kai Cui

**Affiliations:** a Department of Interventional Radiology, Inner Mongolia Forestry General Hospital, The Second Clinical Medical School of Inner Mongolia University for The Nationalities, Yakeshi, Inner Mongolia, China; b Department of Radiology, The First Clinical Medical College, Fujian University of Traditional Chinese Medicine, Fuzhou, China; c Department of Cardiology, Inner Mongolia Forestry General Hospital, The Second Clinical Medical School of Inner Mongolia University for The Nationalities, Yakeshi, Inner Mongolia, China; d Department of Gynaecology and Obstetrics, Inner Mongolia Forestry General Hospital, The Second Clinical Medical School of Inner Mongolia University for The Nationalities, Yakeshi, Inner Mongolia, China.

**Keywords:** dose–response analysis, endometrial cancer, folate intake, meta-analysis

## Abstract

**Background:**

The relationship between folate intake and risk of endometrial cancer (EC) is debatable. The goal of this study was to examine the relationship between folate consumption and EC and then conduct a dose–response analysis in accordance with this.

**Methods:**

Up until February 1, 2024, we conducted a thorough search using PubMed, EMBASE, the Cochrane Library, and Web of Science. Stata 14 software was used to analyze the findings of the article. The study protocol was registered in PROSPERO (CRD42024505943), and the meta-analysis was conducted in accordance with PRISMA guidelines.

**Results:**

Nine case-control studies and 6 cohort studies were included, comprising 379,570 participants and 8660 EC cases. The highest level of folate consumption was associated with a 10% reduction in the occurrence of EC (relative risk [RR] = 0.90, 95% confidence intervals [CIs]: 0.78–1.05, *I*^2^ = 63.2%) compared to the lowest level of intake. The association exhibited a statistically significant linear trend (*P* = .231), with a combined RR of 0.974 (95% CI: 0.968–0.981) for each daily intake of 50 µg folate.

**Conclusion:**

Folate intake may reduce the risk of EC.

## 1. Introduction

Endometrial cancer (EC), as a common cancer in the female reproductive system, ranks second and third in terms of new cases and deaths among gynecological malignancies, respectively.^[1]^ According to statistics, the number of new cases and deaths of EC worldwide in 2020 was 417,367 and 97,370, respectively.^[1]^ Even with advancements in imaging technology and anticancer drugs, EC is frequently discovered late, and its prognosis is not ideal.^[2]^ Age, estrogen use, obesity, early menarche, late menopause, and lifestyle are among the most common risk factors for EC.^[3]^ Developing effective prevention strategies and reducing its severity can be achieved by identifying the risk factors for EC.

Folate, also known as vitamin B9, is a nutrient that may influence cancer formation because it is a 1-carbon unit donor that aids the metabolism of nucleic and amino acids. In vitro evidence substantiating the antitumor properties of folate includes its participation in synthesizing purines and pyrimidines, facilitating DNA methylation, and significantly contributing to DNA repair.^[4]^ According to epidemiological studies, folate can reduce the incidence of colorectal, esophageal, bladder, and pancreatic cancers.^[5]^ Over the past 30 years, several studies have examined the relationship between folate intake and EC. While some studies have indicated that folate intake can lower the risk of EC,^[6]^ many other studies have found no statistically significant relationship between folate intake and the risk of EC.^[7]^ As such, the relationship between folate intake and EC remains controversial. A meta-analysis is necessary to examine the relationship between folate intake and EC risk in greater detail.

## 2. Materials and methods

### 2.1. Search strategy

From their founding until February 1, 2024, we thoroughly searched PubMed, EMBASE, Cochrane Library, and Web of Science databases. In addition to folate, vegetables, fruits, and diet were also employed as search keywords to prevent missing literature that satisfied the standards during retrieval, as folate intake is frequently 1 of the dietary elements under investigation. The fundamental steps in the meta-analysis retrieval process were as follows: (Folic Acid) OR (Vitamin M) OR (Vitamin B9) OR (Folate) OR (Die) OR (Vegetables) OR (Fruit) AND (Endometrial Neoplam) OR (Endometrial Carcinoma) OR (EC). The comprehensive search strategy was included in Table S1, Supplemental Digital Content, http://links.lww.com/MD/N614. This meta-analysis was conducted according to PRISMA guidelines (registration number: CRD40202505943).^[8]^

## 3. Inclusion and exclusion criteria

The inclusion criteria for this study were as follows: The study type was case-control or cohort studies, the relationship between folate intake (comprising total folate intake and dietary folate intake) and EC risk was investigated. Total folate intake encompasses all folate obtained from both food sources and supplementary supplements, whereas dietary folate intake specifically refers to folate obtained solely from food sources, the study furnished folate intake as exposure and EC incidence as outcome, the diagnostic information for EC was acquired through medical diagnosis, self-reporting, pathological diagnosis, medical records, and cancer registration, to assess the association between folate consumption and the EC, the study provided a relative risk (RR), odds ratio (OR), hazard ratio (HR), and 95% confidence intervals (CIs). The following were the criteria for exclusion, the research was published in a language other than English, the article categories consisted of case reports, meta-analyses, editorials, animal experiments, and reviews, the research contained insufficient data.

Two researchers (DW and JL) conducted independent literature reviews using the aforementioned inclusion and exclusion criteria. Disagreements that did arise throughout the screening process were be deliberated and analyzed collaboratively by 2 researchers. When 2 researchers could not reach an agreement, a third researcher (YP) addressed the issue.

## 4. Data extraction

The extracted data for each study comprised the following information: first author, publication year, study location, study design, sample size (number of EC cases and subjects), diet assessment, various levels of folate intake and their corresponding OR values, RR values, HR values, and their respective 95% CI. Additionally, the data included information on the adjusted confounding factors and quality scores. Data were individually extracted by a researcher (ML).

## 5. Quality assessment

To assess the quality of case-control and cohort studies, we employed the Newcastle-Ottawa Scale (NOS), which comprises three primary categories: population selection, intergroup comparability, and exposure/outcome assessment.^[9]^ The scale has a maximum rating of 9 points. Studies with ratings below 4, between 5 and 6, and between 7 and 9 were classified as poor, medium, and high, respectively.

## 6. Data analysis

Given the uncommon occurrence of EC, the OR was approximately equivalent to the RR.^[10]^ Consequently, the RR values and their 95% CIs were consistently utilized as point estimates in this study. The heterogeneity of the estimated values of the merged outcomes was assessed using the *I*^2^ statistic.^[11]^ With minimal to moderate heterogeneity between studies, a fixed effect model (inverse variance method) is typically employed when *I*^2^ < 50%. When *I*^2^ > 50%, moderate to severe heterogeneity exists between studies; therefore, a random-effects model (Mantel-Haenszl method) is required.

Subgroup analysis was conducted if the study exhibited substantial heterogeneity. This involves stratifying the study area, study type, and adjusted confounding factors and assessing whether the subgroup analyses can provide an explanation for the observed heterogeneity. Three methods were employed to identify publication bias in the study: funnel plot,^[12,13]^ Egger regression asymmetry test,^[14]^ and Begg rank correlation test.^[15]^ In the beginning, visually assess the symmetry of the dispersed points within the funnel plot. A study may have no publication bias if the dispersed points have a symmetrical distribution. Furthermore, Egger regression asymmetry test and Begg rank correlation test were implemented to quantify publication bias. Statistically substantial publication bias was indicated in the study when *P* < .05. Additionally, for the sensitivity analysis, we systematically removed each included study and assessed the impact on the overall results by examining the estimated merging points of the remaining studies.

Finally, we employed the methods advised by Greenland and Longnecker to examine the possible dose–response association between folate consumption and EC.^[16]^ To conduct a dose–response analysis, each included study was divided into at least 3 folate intake groups and provided the following 4 sets of data: folate intake levels in each group, number of EC cases corresponding to folate intake levels in each group, number of people per/year in each group, point estimates for each group, and their respective 95% CIs. To assess whether the dose–response association between folate intake and EC is linear or nonlinear, we used a restricted cubic spline function with 4 nodes (5%, 35%, 65%, and 95%).^[17]^ A linear dose–response association existed between folate consumption and EC when *P* < .05, but a nonlinear relationship otherwise. We calculated folate consumption for the original study group by taking the midpoint of the upper and lower bounds of the interval. For the upper open interval, we multiplied the endpoint by 1.5. For the lower open interval, we divided the interval endpoint by 1.5.

All statistical analyses were performed using Stata 14.0 (StataCorp, College Station, TX, USA). *P* < .05 was considered statistically significant.

## 7. Results

### 7.1. Literature search

**Figure 1.**
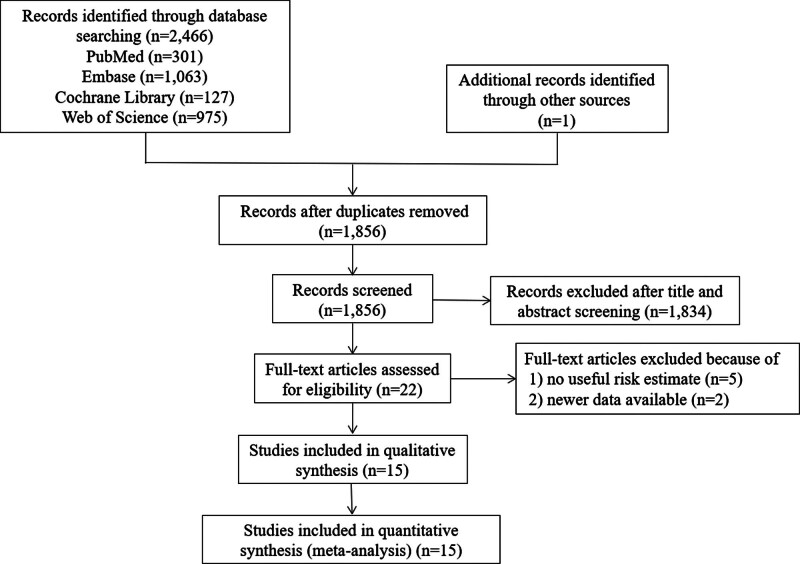
Literature search and screening process.

Following an initial search, a comprehensive compilation of 2466 documents was extracted from the 4 databases. An additional record was acquired via other resources. The initial search yielded 2467 scholarly articles. Following the elimination of duplicate studies, 1856 records remained. After reviewing their titles and abstracts, a total of 1834 items were excluded, leaving 22 studies. 2 articles with outdated data and 5 that failed to provide point estimates were excluded after thorough examination of the entire text. Finally, 15 studies were included in the meta-analysis (Fig. 1).

## 8. Study characteristics

Table 1 presented the research characteristics of this study. Of the 15 included studies, 6 were cohort studies^[19,23,26,28–30]^ and 9 were case-control studies.^[6,7,18,20–22,24,25,27]^ Seven studies were conducted in USA,^[6,7,20,24,26,28,30]^ 5 studies in Canada^[18,19,23,25,29]^ 1 in Mexico,^[21]^ 1 in Europe,^[27]^ and 1 in China.^[22]^ With 379,570 participants, this study documented 8660 cases of EC. To assess folate intake, 15 studies employed food frequency questionnaires (FFQ). The majority of studies have considered or controlled for confounding variables that influence EC, such as age, BMI, estrogen use, contraceptive use, total energy consumption, parity, smoking, education, age at menarche, history of diabetes, and menopause. Case-control studies and cohort studies obtained mean quality assessment scores of 8.00 (SD = 0.71) and 7.67 (SD = 1.03), respectively (Table 1, Table S2, Supplemental Digital Content, http://links.lww.com/MD/N614).

**Table 1:** Characteristics of included studies.

Author	Region	Study type	Case/ subjects	Diet assessment	Intake measurement	RR (95% CI)	Adjustment	Quality score
Potischman et al^[7]^ (1993)	USA	Case-control	399/296	Validated FFQ (60 items)	Folate-rich foods Q1 (<6.4 times/wk) Q2 (6.4–10.7 times/wk) Q3 (10.8–15.9 times/wk) Q4 (>15.9 times/wk)	1.0 0.8 (0.5–1.3) 0.9 (0.5–1.4) 0.9 (0.6–1.6)	Age, BMI, used hormone replacement therapy, ever-contraceptive usage, parity, smoking, education, total energy	8
Jain et al^[18]^ (2000)	Canada	Case-control	552/562	Validated FFQ (142 items)	Q1 (low) Q2 Q3 Q4 (high)	1.0 1.17 (0.83–1.65) 0.88 (0.62–1.26) 0.96 (0.67–1.36)	Age, total energy, body weight, smoking, history of diabetes, ever-contraceptive usage, used hormone replacement therapy, education, parity, age at menarche	8
Jain et al^[19]^ (2000)	Canada	Cohort	221/56,837	Validated FFQ (86 items)	Q1 (low) Q2 Q3 Q4 (high)	1.00 0.95 (0.62–1.44) 1.44 (0.77–1.70) 1.18 (0.79–1.74)	Age, total energy, body weight, smoking, history of diabetes, ever-contraceptive usage, used hormone replacement therapy, education, parity, age at menarche	6
McCann et al^[6]^(2000)	USA	Case-control	232/639	Validated FFQ (172 items)	Q1 (<296 μg/d) Q2 (297–256 μg/d) Q3 (357–448 μg/d) Q4 (>448 μg/d)	1.0 0.5 (0.3–0.9) 0.7 (0.4–1.1) 0.4 (0.2–0.7)	Age, education, BMI, diabetes, hypertension, smoking, age at menarche, parity, ever-contraceptive usage, menopause status, used hormone replacement therapy, total energy	9
Paynter et al^[20]^ (2004)	USA	Case-control	201/603	Validated FFQ (no mention)	<400 μg/d ≥400 μg/d	1.00 0.74 (0.52–1.07)	Age, alcohol, total energy	7
Martinez et al^[21]^ (2005)	Mexico	Case-control	85/629	Validated FFQ (116 items)	≤197 μg/d 198–321 μg/d ≥322 μg/d	1.00 0.84 (0.44–1.61) 1.04 (0.48–2.23)	Age, total energy intake, parity, BMI, physical activity, and history of diabetes	7
Xu et al^[22]^ (2007)	China	Case-control	1204/1212	Validated FFQ (71 items)	Q1 (low) Q2 Q3 Q4	1.0 0.8 (0.7–1.1) 0.6 (0.5–0.8) 0.6 (0.4–0.7)	Age, education, menopausal status, history of diabetes, alcohol consumption, BMI, physical activity, total energy, total animal food intake, and total fruit and vegetable intake	8
Kabat et al^[23]^ (2008)	Canada	Cohort	426/49,654	Validated FFQ (86 items)	<237 μg/d 237 to <281 μg/d 281 to <321 μg/d 321 to <374 μg/d 374+	1.00 0.91 (0.65–1.26) 1.07 (0.78–1.48) 1.12 (0.81–1.54) 0.79 (0.55–1.13)	Age, BMI, education, menopausal status, parity, age at menarche, ever-contraceptive usage, used hormone replacement therapy, and total energy, calcium, and raw vegetables	8
Yeh et al^[24]^ (2009)	USA	Case-control	541/541	Validated FFQ (44 items)	Q1 (≤288 μg/d) Q2 (289–371 μg/d) Q3 (372–473 μg/d) Q4 (≥474 μg/d)	1.00 0.74 (0.51–1.07) 0.69 (0.46–1.01) 0.57 (0.36–0.91)	Age, BMI, used hormone replacement therapy, smoking, lifetime duration of menstruation, and total energy	8
Biel et al^[25]^ (2011)	Canada	Case-control	506/981	Validated FFQ (124 items)	≤277.6 μg/d >277.6 to ≤322.5 μg/d 322.5 to ≤377.6 μg/d >377.6 to ≤851.3 μg/d	1.00 0.80 (0.57–1.12) 1.14 (0.83–1.57) 1.18 (0.85–1.63)	Age, total energy, nutrient-specific supplement use, age at menarche, BMI, parity, education, hyperteosion history, ever-contraceptive usage, used hormone replacement therapy, menopausal status, and alcohol consumption	9
Uccella et al^[26]^ (2011)	USA	Cohort	471/23,356^*^ 71 /23,356^†^	Validated FFQ (126 items)	Type I endometrial cancer: 43.5 to 250.1 μg/d 250.2 to 348.6 μg/d 348.7 to 560.9 μg/d >560.9 μg/d Type II endometrial cancer: 43.5 to 250.1 μg/d 250.2 to 348.6 μg/d 348.7 to 560.9 μg/d >560.9 μg/d	1.00 0.85 (0.64–1.13) 1.08 (0.81–1.44) 1.00 (0.76–1.32) 1.00 0.93 (0.45–1.95) 0.97 (0.44–2.12) 1.71 (0.87–3.35)	Age, total energy, BMI, waist-to-hip ratio, history of diabetes, hypertension, age at menopause, used hormone replacement therapy, smoking, and alcohol use	8
Tavani et al^[27]^ (2012)	Italy and Switzerland	Case-control	454/1366	Validated FFQ (78 items)	Q1 Q2 Q3 Q4	1.00 1.10 (0.77–1.57) 0.79 (0.53–1.18) 1.06 (0.67–1.67)	Age, sex, study center, year of interview, education, alcohol drinking, smoking, BMI, total energy, and physical activity at work	8
Liu et al^[28]^ (2013)	USA	Cohort	788/121,700	Validated FFQ (no mention)	Q1:275.8 μg/d Q2:402.0 μg/d Q3:509.7 μg/d Q4:623.8 μg/d Q5:794.0 μg/d	1.00 1.17 (0.93–1.48) 1.11 (0.87–1.41) 1.19 (0.94–1.51) 1.10 (0.87–1.41)	Age, calendar year, smoking, BMI, race, age at menarche, ever-contraceptive usage, menopausal status, used hormone replacement therapy, and parity	7
Arthur et al^[29]^ (2019)	Canada	Cohort	180/2606	Validated FFQ (166 items)	≤389.5 μg/d 389.5–487.2 μg/d 487.3–614.9 μg/d >614.9 μg/d	1.00 1.02 (0.68–1.53) 0.86 (0.56–1.31) 0.52 (0.29–0.93)	Education, smoking, alcohol intake, BMI, total energy, physical activity, age at menarche, parity, breastfeeding, menopausal status, HRT use, ever-contraceptive usage, family history	9
Lu et al^[30]^ (2019)	USA	Cohort	2329/114,414	Validated FFQ (no mention)	Type I endometrial cancer: Q1 Q2 Q3 Q4 Q5 Type II endometrial cancer: Q1 Q2 Q3 Q4 Q5	1.00 1.09 (0.94–1.27) 1.07 (0.92–1.24) 1.10 (0.94–1.27) 1.15 (0.99–1.34) 1.00 1.07 (0.66–1.75) 1.16 (0.72–1.87) 0.82 (0.49–1.40) 1.13 (0.69–1.83)	Age, BMI, smoking, ever-contraceptive usage, used hormone replacement therapy, and total energy	8

## 9. Overall analysis and dose–response analysis

**Figure 2.**
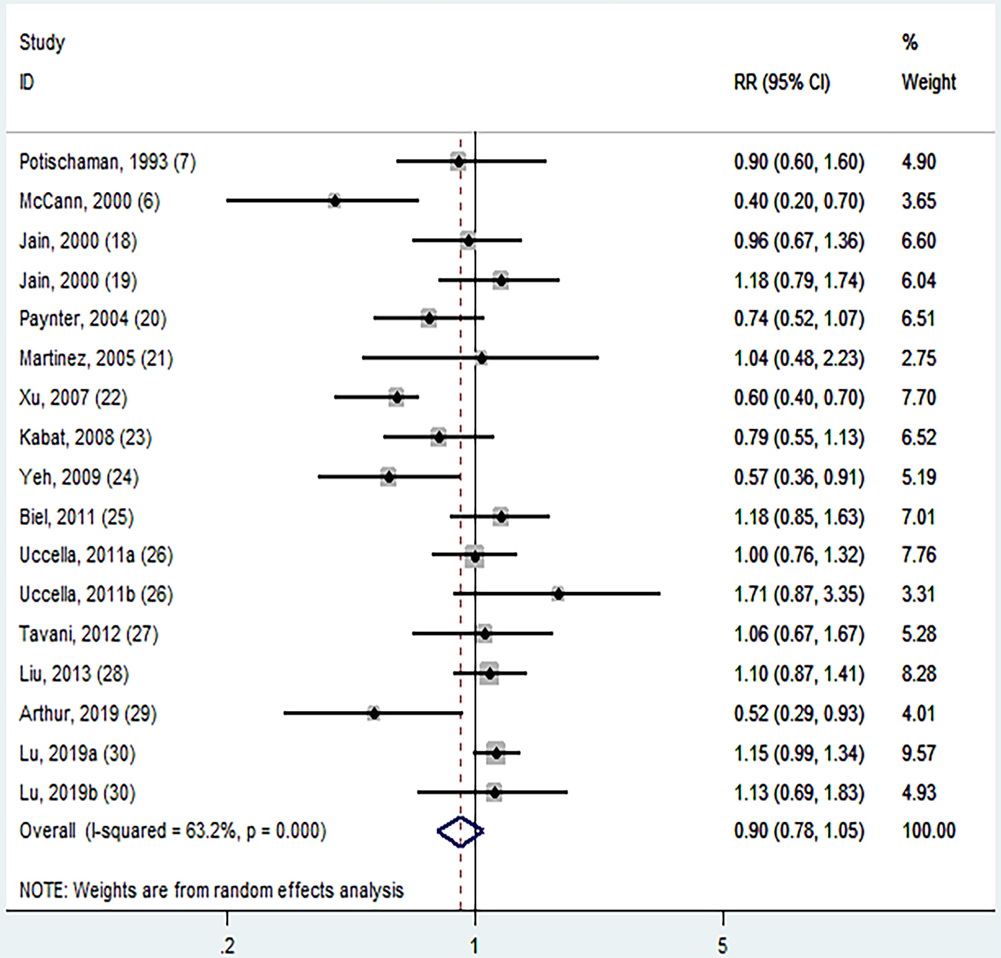
Forest plot of the association between folate intake and EC.

**Figure 3.**
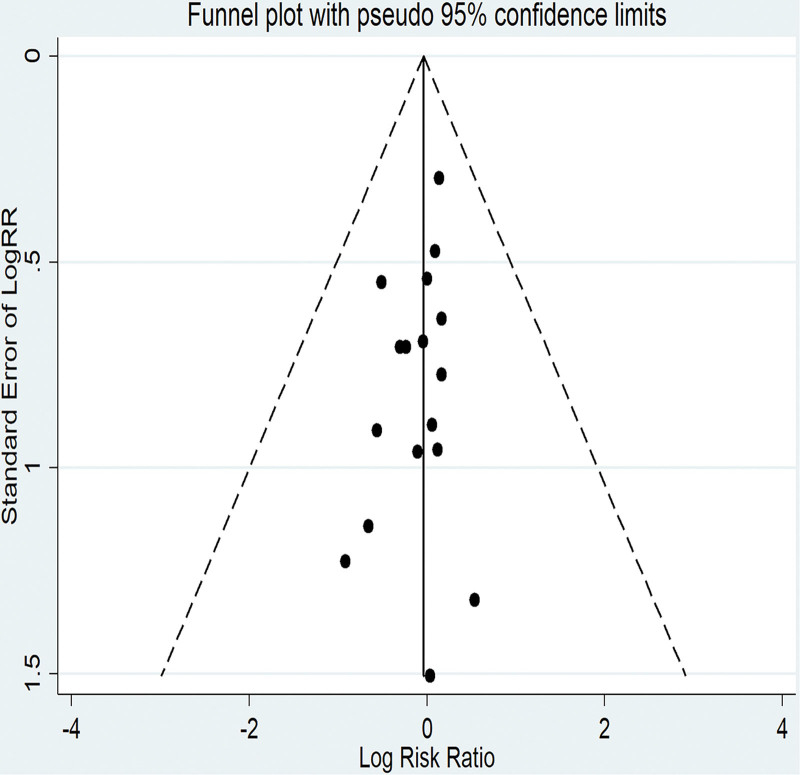
The result of funnel plot.

The association between folate intake and EC was depicted in Figure 2. The RR values included in the study ranged from McCann et al’s 0.40 (95% CI: 0.20–0.70)^[6]^ to Uccella et al’s 1.71 (95% CI: 0.87–3.35).^[26]^ The highest group folate intake can reduce the incidence of EC by 10% (RR = 0.90; 95% CI: 0.78–1.05) compared to the lowest group intake. A random effects model was employed due to the moderate heterogeneity (*I*^2^ = 63.2%, *P* = .0000) observed in the summary study. Visual examination of the funnel plot revealed that the scattered points at both extremities were symmetrically distributed, and there was no discernible publication bias (Fig. 3). Concurrently, both Egger test (*P* = .132) (Figure S1, Supplemental Digital Content, http://links.lww.com/MD/N614) and Begg test (*P* = .246) (Figure S2, Supplemental Digital Content, http://links.lww.com/MD/N614) failed to identify any indications of publication bias.

**Figure 4.**
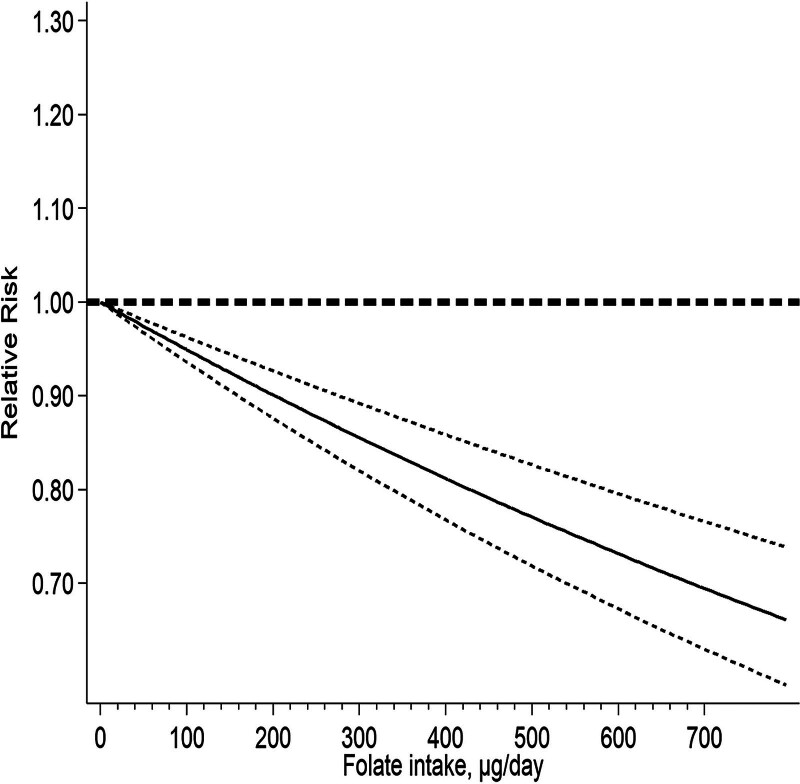
RR for endometrial cancer by doses of folate consumption in light of the results of the dose–response meta-analyses.

The dose–response analysis results were illustrated in Figure 4. Research has shown that the intake of folate may reduce the risk of EC (RR = 0.90; 95% CI: 0.78–1.05). The risk of EC is decreased by 2.57% with each daily addition of 50 micrograms of folate (RR = 0.974, 95% CI: 0.968–0.981, *P* = .231).

## 10. Subgroup analysis and sensitivity analysis

Table 2 displayed the subgroup analysis results by study type, area, and adjustment factors. When stratified by study type, folate intake reduced the incidence of EC in case-control studies (RR = 0.80, 95% CI: 0.70–0.91) but not in cohort studies (RR = 1.07, 95% CI: 0.97–1.19). When stratified by research region, studies from China suggest that folate intake can reduce the risk of EC (RR = 0.60, 95% CI: 0.45–0.79), but studies from the USA (RR = 1.02, 95% CI: 0.92–1.12), Canada (RR = 0.90, 95% CI: 0.81–1.14), Mexico (RR = 1.04, 95% CI: 0.48–2.24), and Europe (RR = 1.06, 95% CI: 0.67–1.67) do not believe folate intake can reduce the risk of EC. Adjustment for BMI, contraceptive use, hormone replacement therapy, parity, smoking, education, total energy intake, age at menarche, diabetes history, and menopause had no significant effect on the risk of EC in the subgroup analysis.

**Table 2:** Subgroup analysis of folate intake with the risk of EC.

	No of studies	Summary RR	95% Cl	*I*^2^ (%)	*P*-value
Overall	15	0.90	(0.78–1.02)	63.2	.000
Study type					
Case-control	9	0.80	(0.70–0.91)	59.2	.012
Cohort	6	1.07	(0.97–1.19)	41.2	.103
Region					
USA	7	1.02	(0.92–1.12)	65.4	.003
Canada	5	0.96	(0.81–1.14)	49.8	.093
Mexico	1	1.04	(0.48–2.24)		
China	1	0.60	(0.45–0.79)		
Italy and Switzerland	1	1.06	(0.67–1.67)		
Adjustment for confounders					
BMI					
Yes	12	0.99	(0.91–1.08)	61.1	.001
No	3	0.76	(0.60–0.96)	65.1	.057
Ever-contraceptive usage					
Yes	9	1.04	(0.94–1.14)	55.3	.017
No	6	0.81	(0.70–0.94)	61.5	.016
Hormone replacement therapy					
Yes	10	1.04	(0.95–1.14)	52.8	.016
No	5	0.71	(0.59–0.85)	37.9	.168
Parity					
Yes	8	0.99	(0.86–1.13)	54.9	.030
No	7	0.95	(0.86–1.05)	71.2	.001
Smoking					
Yes	10	1.03	(0.94–1.13)	57.5	.007
No	5	0.79	(0.68–0.93)	60.6	.038
Education					
Yes	8	0.88	(0.78–1.00)	55.1	.023
No	7	1.02	(0.93–1.13)	69.2	.002
Total energy intake					
Yes	12	0.98	(0.89–1.07)	64.0	.001
No	3	0.91	(0.76–1.09)	70.9	.032
Age at menarche					
Yes	7	0.94	(0.85–1.05)	70.6	.001
No	8	0.99	(0.87–1.11)	58.8	.013
History of diabetes					
Yes	10	0.99	(0.90–1.09)	64.7	.002
No	5	0.90	(0.78–1.05)	64.7	.015
Menopause status					
Yes	10	1.02	(0.92–1.12)	45.0	.045
No	5	0.86	(0.75–0.99)	79.9	.001

## 11. Discussion

The current meta-analysis included 15 observational studies (nine case-control studies and 6 cohort studies), revealing a boundary-negative connection between folate consumption and EC (RR = 0.90, 95% CI: 0.78–1.05). Du et al^[31]^ conducted the first meta-analysis on the association between folate intake and EC. We performed an additional dose–response analysis on this premise. According to our findings, taking an extra 50 µg of folate daily lowered the risk of EC by 2.57% (RR = 0.974, 95% CI: 0.968–0.981, *P* = .231).

Our research results were primarily based on case-control studies, as evidenced by the summary analysis of case-control studies that suggested that folate intake could lower the risk of EC (RR = 0.80, 95% CI: 0.70–0.91). However, this relationship was not significant (RR = 1.07, 95% CI: 0.97–1.19) in cohort studies. The correlation between China (RR = 0.60, 95% CI: 0.45–0.79), Canada (RR = 0.96, 95% CI: 0.81–1.14), and the USA (RR = 1.02, 95% CI: 0.92–1.12) was higher when stratified analysis by study area than between Mexico (RR = 1.04, 95% CI: 0.48–2.24) and Europe (RR = 1.06, 95% CI: 0.67–1.67). This suggests that regional variations could account for some of the moderate heterogeneity observed in the study findings. In addition, 4 of the 7 studies included in the USA were case-control studies,^[6,7,20,24]^ which could account for the internal heterogeneity of the studies. A subgroup analysis of the significant confounding variables that influenced the incidence of EC was also performed. These variables included BMI, contraceptive use, hormone replacement therapy, parity, smoking, education, total energy intake, age at menarche, history of diabetes, and menopause.

There is no denying that the study population could be a source of heterogeneity. To make the study more thorough, we did not establish exclusion criteria for the study population. Two studies examined the association between vitamin B intake and the risk of common tumors in women.^[23,29]^ 3 studies examined the link between carbon metabolism EC risk.^[26,28,30]^ 3 studies examined the association between MTHFR gene types and EC risk.^[20,22,28]^ There are several mechanisms by which folate can influence on tumor development: regulates DNA methylation process. By providing methyl groups to homocysteine, the active component of folate, known as 5-methyltetrahydrofolate, facilitates the synthesis of methionine.^[32]^ S-adenosylmethionine (SAM) is produced through the methylation of methionine. SAM methylates cytosine through DNA methyltransferase activity, thereby regulating gene transcription and expression^[33]^; regulate the integrity of DNA. A reduction in folate levels results in a depletion of 5,10-methylenetetrahydrofolate levels within cells, which subsequently inhibits the methylation of deoxyuridine monophosphate (dUTP) to deoxythymidine triphosphate (dTTP) in a proportional fashion.^[32]^ Temporary single-strand breaks may result from DNA polymerase incorporating uracil into DNA in response to decreased dUMP/dTMP ratio.^[33]^ Chromosomal breaking can transpire due to 2 gaps oriented in opposing directions, potentially leading to the progression of malignancies.^[33]^ Folic acid, or pteroylglutamic acid, is derived from fortified foods, supplements, and medications. It is an artificially produced version of folate. Folate, scientifically referred to as 5-methyltetrahydrofolate, is derived from green leafy vegetables and is a biologically active form of folate acquired through the processes of reduction and methylation. Our meta-analysis included 8 studies that reported dietary folate intake and 7 studies that reported total folate intake. Our research indicates that consuming dietary folate can lower the risk of EC (RR = 0.81, 95% CI: 0.71–0.93), providing evidence that aligns with the aforementioned theoretical foundation.

Dihydrofolate reductase converts folic acid into tetrahydrofolate. Exceeding 400 mg of folic acid can completely saturate dihydrofolate reductase, resulting in residual folic acid.^[4]^ This leftover folic acid has the potential to cause cancer.^[4]^ Simultaneously, increased folic acid supplementation may have a tumor-promoting effect on pre-tumor lesions.^[34]^ Our analysis revealed that total folate intake increased the incidence of EC (RR = 1.05, 95% CI: 0.95–1.16), although this association was not statistically significant. However, total folate intake included both folic acid supplementation and dietary folate intake. Therefore, our results may be influenced by both the negative and positive effects of folate on folic levels. Prospective research is needed to assess the association between folic acid supplementation and EC.

The p53 gene mutation is a common feature of type II EC, with a mutation rate ranging from 71% to 85%.^[35,36]^ The P53 mutation rate in type I EC is barely one-third.^[35,36]^ In contrast, p53 mutations are associated with abnormal cell proliferation and DNA damage. As a result, different EC subtypes may react differently to folate intake. Nonetheless, our meta-analysis included only 2 studies that examined the relationship between folate consumption and the risk of EC in different subtypes.^[26,30]^ Consequently, additional studies are needed to determine the relationship between folate consumption and the risk of different EC subtypes.

The benefit of our study is that we performed a further dose–response analysis to show a linear reduction in the risk of EC linked to folate consumption. Furthermore, subgroup analysis was performed to elucidate the moderate heterogeneity observed in this study. Our research does, however, have several limitations. First, to determine the frequency of dietary consumption and folate intake, all the included studies employed the FFQ, a tool for food nutrition evaluation. However, numerous studies have adjusted the FFQ to better reflect the variety of food in the study location, leading to variations in the items included in various studies. There were 44 items in some studies and 172 in others. The FFQ, however, depends on an individual’s accurate dietary recall. As a result, it is possible to overestimate or underestimate folate consumption, which could have impacted the findings of this study. Second, although we performed a subgroup analysis of the researched regions, 12 out of 15 studies came from North America. Thus, more studies on the association between folate intake and EC risk need to be conducted outside of North America. Third, case-control and cohort studies were the 2 categories of observational studies included in this research. However, recall, information, and confounding bias affect impact case-control studies. Fourth, due to the limited sample size we included in the study, we were unable to draw accurate conclusions. Finally, most clinical doctors will adjust their treatment strategies based on the molecular subtypes of EC patients. However, the studies included in this meta-analysis lack data on molecular subtypes and have not been able to further evaluate the risk of folate intake and different EC molecular subtypes.

In conclusion, our findings suggest that folate intake may be related to a lower EC risk. According to the results of dose–response research, taking an extra 50 µg of folate per day can reduce the risk of EC by 2.57%.

## Author contributions

**Conceptualization:** Jiaye Long, Du Wang, Yingrong Pang.

**Data curation:** Du Wang.

**Formal analysis:** Yingrong Pang.

**Investigation:** Miyang Yang, Yingrong Pang.

**Methodology:** Miyang Yang.

**Software:** Meiqiong Li.

**Supervision:** Shuxin Qin.

**Writing – original draft:** Jiaye Long.

**Writing – review & editing:** Jiaye Long, Kai Cui.

## Supplementary Material


