# Supplementary material for: Folate intake and the risk of endometrial cancer: A dose–response meta-analysis
Source: Medicine (Baltimore). 2024 Sep 20;103(38):e39775. doi: 10.1097/MD.0000000000039775 (PMC11419418; doi:10.1097/MD.0000000000039775)
Supplement: Supplementary file 1 [file medi-103-e39775-s001.docx]

Supplementary Figure 1. The Result of of the Egger's test.


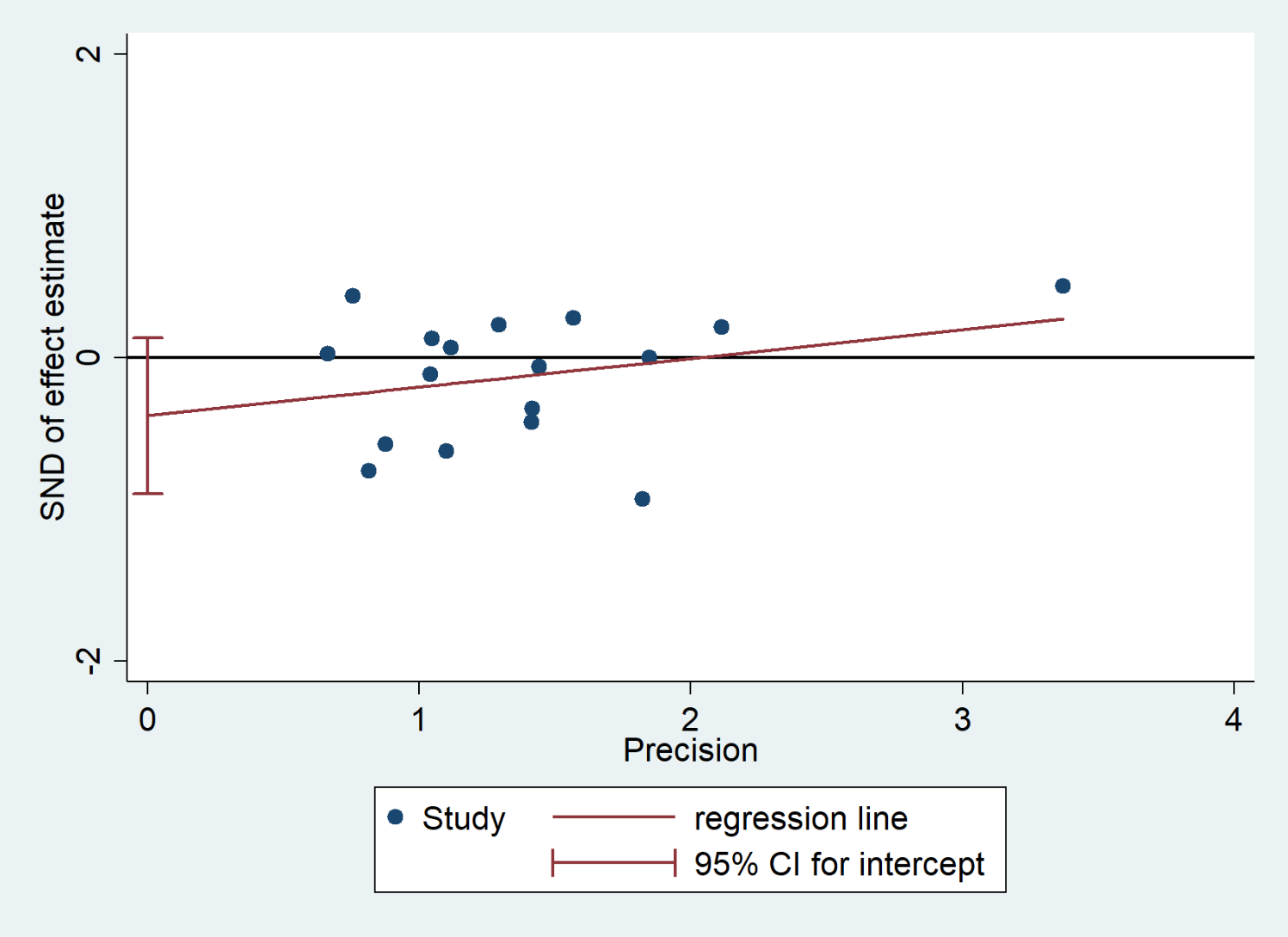


Supplementary Figure 2. The Result of of the Begg's test.


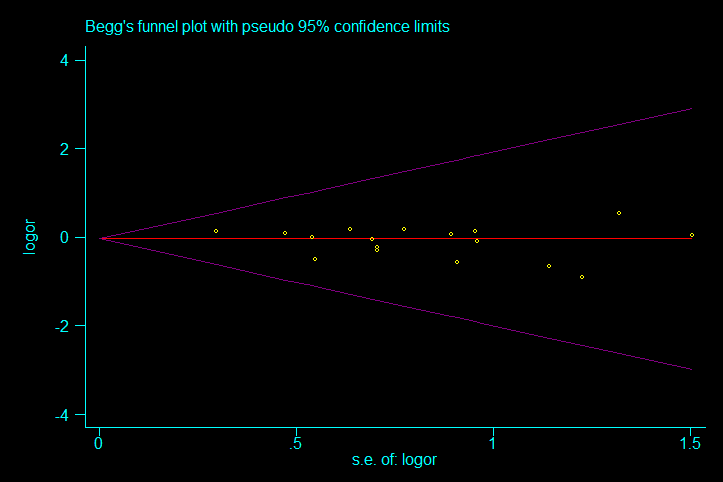


Supplementary Table 1. Detailed search strategy

| **PubMed** |  |  |
| --- | --- | --- |
| Search | Query | Results |
| #1 | "Folic Acid"[MeSH Terms] | 42,359 |
| #2 | "vitamin m"[Title/Abstract] OR "vitamin b9"[Title/Abstract] OR "b9 vitamin"[Title/Abstract] OR "pteroylglutamic acid"[Title/Abstract] OR ("Folic"[All Fields] AND "acid monopotassium salt"[Title/Abstract]) OR ("Folic"[All Fields] AND "acid monosodium salt"[Title/Abstract]) OR ("Folic"[All Fields] AND "acid potassium salt"[Title/Abstract]) OR ((("folic acid"[MeSH Terms] OR ("Folic"[All Fields] AND "Acid"[All Fields]) OR "folic acid"[All Fields]) AND "DL"[All Fields]) AND "isomer"[Title/Abstract]) OR "Folvite"[Title/Abstract] OR "Folacin"[Title/Abstract] OR "Folate"[Title/Abstract] OR ((("folic acid"[MeSH Terms] OR ("Folic"[All Fields] AND "Acid"[All Fields]) OR "folic acid"[All Fields]) AND "D"[All Fields]) AND "isomer"[Title/Abstract]) OR ((("folic acid"[MeSH Terms] OR ("Folic"[All Fields] AND "Acid"[All Fields]) OR "folic acid"[All Fields]) AND ("calcium"[MeSH Terms] OR "calcium"[All Fields] OR "calciums"[All Fields] OR "calcium s"[All Fields]) AND ("sodium chloride"[MeSH Terms] OR ("Sodium"[All Fields] AND "chloride"[All Fields]) OR "sodium chloride"[All Fields] OR "Salt"[All Fields])) AND "1 1"[Title/Abstract]) OR ("Folic"[All Fields] AND "acid sodium salt"[Title/Abstract]) | 31,384 |
| #3 | "Folic Acid"[MeSH Terms] OR ("vitamin m"[Title/Abstract] OR "vitamin b9"[Title/Abstract] OR "b9 vitamin"[Title/Abstract] OR "pteroylglutamic acid"[Title/Abstract] OR ("Folic"[All Fields] AND "acid monopotassium salt"[Title/Abstract]) OR ("Folic"[All Fields] AND "acid monosodium salt"[Title/Abstract]) OR ("Folic"[All Fields] AND "acid potassium salt"[Title/Abstract]) OR ((("Folic Acid"[MeSH Terms] OR ("Folic"[All Fields] AND "Acid"[All Fields]) OR "Folic Acid"[All Fields]) AND "DL"[All Fields]) AND "isomer"[Title/Abstract]) OR "Folvite"[Title/Abstract] OR "Folacin"[Title/Abstract] OR "Folate"[Title/Abstract] OR ((("Folic Acid"[MeSH Terms] OR ("Folic"[All Fields] AND "Acid"[All Fields]) OR "Folic Acid"[All Fields]) AND "D"[All Fields]) AND "isomer"[Title/Abstract]) OR ((("Folic Acid"[MeSH Terms] OR ("Folic"[All Fields] AND "Acid"[All Fields]) OR "Folic Acid"[All Fields]) AND ("calcium"[MeSH Terms] OR "calcium"[All Fields] OR "calciums"[All Fields] OR "calcium s"[All Fields]) AND ("sodium chloride"[MeSH Terms] OR ("Sodium"[All Fields] AND "chloride"[All Fields]) OR "sodium chloride"[All Fields] OR "Salt"[All Fields])) AND "1 1"[Title/Abstract]) OR ("Folic"[All Fields] AND "acid sodium salt"[Title/Abstract])) | 57,320 |
| #4 | "Diet"[MeSH Terms] | 334,432 |
| #5 | "Vegetables"[MeSH Terms] | 38,025 |
| #6 | "Fruit"[MeSH Terms] | 128,654 |
| #7 | ((("Folic Acid"[MeSH Terms] OR ("vitamin m"[Title/Abstract] OR "vitamin b9"[Title/Abstract] OR "b9 vitamin"[Title/Abstract] OR "pteroylglutamic acid"[Title/Abstract] OR ("Folic"[All Fields] AND "acid monopotassium salt"[Title/Abstract]) OR ("Folic"[All Fields] AND "acid monosodium salt"[Title/Abstract]) OR ("Folic"[All Fields] AND "acid potassium salt"[Title/Abstract]) OR ((("Folic Acid"[MeSH Terms] OR ("Folic"[All Fields] AND "Acid"[All Fields]) OR "Folic Acid"[All Fields]) AND "DL"[All Fields]) AND "isomer"[Title/Abstract]) OR "Folvite"[Title/Abstract] OR "Folacin"[Title/Abstract] OR "Folate"[Title/Abstract] OR ((("Folic Acid"[MeSH Terms] OR ("Folic"[All Fields] AND "Acid"[All Fields]) OR "Folic Acid"[All Fields]) AND "D"[All Fields]) AND "isomer"[Title/Abstract]) OR ((("Folic Acid"[MeSH Terms] OR ("Folic"[All Fields] AND "Acid"[All Fields]) OR "Folic Acid"[All Fields]) AND ("calcium"[MeSH Terms] OR "calcium"[All Fields] OR "calciums"[All Fields] OR "calcium s"[All Fields]) AND ("sodium chloride"[MeSH Terms] OR ("Sodium"[All Fields] AND "chloride"[All Fields]) OR "sodium chloride"[All Fields] OR "Salt"[All Fields])) AND "1 1"[Title/Abstract]) OR ("Folic"[All Fields] AND "acid sodium salt"[Title/Abstract]))) OR ("Diet"[Mesh])) OR ("Vegetables"[Mesh])) OR ("Fruit"[Mesh]) | 521,921 |
| #4 | "Endometrial Neoplasms"[MeSH Terms] | 26,520 |
| #5 | "endometrial neoplasm"[Title/Abstract] OR "neoplasm endometrial"[Title/Abstract] OR "neoplasms endometrial"[Title/Abstract] OR "endometrial carcinoma"[Title/Abstract] OR "carcinoma endometrial"[Title/Abstract] OR "carcinomas endometrial"[Title/Abstract] OR "endometrial carcinomas"[Title/Abstract] OR "endometrial cancer"[Title/Abstract] OR "cancer endometrial"[Title/Abstract] OR "cancers endometrial"[Title/Abstract] OR "endometrial cancers"[Title/Abstract] OR "endometrium cancer"[Title/Abstract] OR "cancer endometrium"[Title/Abstract] OR "cancers endometrium"[Title/Abstract] OR "cancer of the endometrium"[Title/Abstract] OR "carcinoma of endometrium"[Title/Abstract] OR "endometrium carcinoma"[Title/Abstract] OR "endometrium carcinomas"[Title/Abstract] OR "cancer of endometrium"[Title/Abstract] OR "endometrium cancers"[Title/Abstract] | 31,651 |
| #6 | (((("Folic Acid"[MeSH Terms] OR ("vitamin m"[Title/Abstract] OR "vitamin b9"[Title/Abstract] OR "b9 vitamin"[Title/Abstract] OR "pteroylglutamic acid"[Title/Abstract] OR ("Folic"[All Fields] AND "acid monopotassium salt"[Title/Abstract]) OR ("Folic"[All Fields] AND "acid monosodium salt"[Title/Abstract]) OR ("Folic"[All Fields] AND "acid potassium salt"[Title/Abstract]) OR ((("Folic Acid"[MeSH Terms] OR ("Folic"[All Fields] AND "Acid"[All Fields]) OR "Folic Acid"[All Fields]) AND "DL"[All Fields]) AND "isomer"[Title/Abstract]) OR "Folvite"[Title/Abstract] OR "Folacin"[Title/Abstract] OR "Folate"[Title/Abstract] OR ((("Folic Acid"[MeSH Terms] OR ("Folic"[All Fields] AND "Acid"[All Fields]) OR "Folic Acid"[All Fields]) AND "D"[All Fields]) AND "isomer"[Title/Abstract]) OR ((("Folic Acid"[MeSH Terms] OR ("Folic"[All Fields] AND "Acid"[All Fields]) OR "Folic Acid"[All Fields]) AND ("calcium"[MeSH Terms] OR "calcium"[All Fields] OR "calciums"[All Fields] OR "calcium s"[All Fields]) AND ("sodium chloride"[MeSH Terms] OR ("Sodium"[All Fields] AND "chloride"[All Fields]) OR "sodium chloride"[All Fields] OR "Salt"[All Fields])) AND "1 1"[Title/Abstract]) OR ("Folic"[All Fields] AND "acid sodium salt"[Title/Abstract]))) OR ("Diet"[Mesh])) OR ("Vegetables"[Mesh])) OR ("Fruit"[Mesh])) AND ("endometrial neoplasm"[Title/Abstract] OR "neoplasm endometrial"[Title/Abstract] OR "neoplasms endometrial"[Title/Abstract] OR "endometrial carcinoma"[Title/Abstract] OR "carcinoma endometrial"[Title/Abstract] OR "carcinomas endometrial"[Title/Abstract] OR "endometrial carcinomas"[Title/Abstract] OR "endometrial cancer"[Title/Abstract] OR "cancer endometrial"[Title/Abstract] OR "cancers endometrial"[Title/Abstract] OR "endometrial cancers"[Title/Abstract] OR "endometrium cancer"[Title/Abstract] OR "cancer endometrium"[Title/Abstract] OR "cancers endometrium"[Title/Abstract] OR "cancer of the endometrium"[Title/Abstract] OR "carcinoma of endometrium"[Title/Abstract] OR "endometrium carcinoma"[Title/Abstract] OR "endometrium carcinomas"[Title/Abstract] OR "cancer of endometrium"[Title/Abstract] OR "endometrium cancers"[Title/Abstract]) | 301 |
| **Embase** | | |
| Search | Query | Results |
| #42 | #19 AND #41 | 1,063 |
| #41 | #20 OR #21 OR #22 OR #23 OR #24 OR #25 OR #26 OR #27 OR #28 OR #29 OR #30 OR #31 OR #32 OR #33 OR #34 OR #35 OR #36 OR #37 OR #38 OR #39 OR #40 | 92,767 |
| #40 | 'endometrium cancers':ab,ti | 32 |
| #39 | 'cancer of endometrium':ab,ti | 36 |
| #38 | 'endometrium carcinomas':ab,ti | 28 |
| #37 | 'endometrium carcinoma':ab,ti | 177 |
| #36 | 'carcinoma of endometrium':ab,ti | 106 |
| #35 | 'cancer of the endometrium':ab,ti | 351 |
| #34 | 'cancers, endometrium':ab,ti | 10 |
| #33 | 'cancer, endometrium':ab,ti | 41 |
| #32 | 'endometrium cancer':ab,ti | 306 |
| #31 | 'endometrial cancers':ab,ti | 4,382 |
| #30 | 'cancers, endometrial':ab,ti | 71 |
| #29 | 'cancer, endometrial':ab,ti | 729 |
| #28 | 'endometrial cancer':ab,ti | 34,396 |
| #27 | 'endometrial carcinomas':ab,ti | 3,162 |
| #26 | 'carcinomas, endometrial':ab,ti | 23 |
| #25 | 'carcinoma, endometrial':ab,ti | 183 |
| #24 | 'endometrial carcinoma':ab,ti | 14,446 |
| #23 | 'neoplasms, endometrial':ab,ti | 15 |
| #22 | 'neoplasm, endometrial':ab,ti | 7 |
| #21 | 'endometrial neoplasm':ab,ti | 43 |
| #20 | 'endometrium tumor'/exp | 87,651 |
| #19 | #1 OR #2 OR #3 OR #4 OR #5 OR #6 OR #7 OR #8 OR #9 OR #10 OR #11 OR #12 OR #13 OR #14 OR #15 OR #16 OR #17 OR #18 | 734,230 |
| #18 | 'fruit':ab,ti | 99,988 |
| #17 | 'vegetables':ab,ti | 57,990 |
| #16 | 'diet':ab,ti | 53,6895 |
| #15 | 'folic acid, sodium salt':ab,ti | 0 |
| #14 | 'folic acid, calcium salt (1:1)':ab,ti | 0 |
| #13 | 'folic acid, (d)-isomer':ab,ti | 0 |
| #12 | 'folate':ab,ti | 40,186 |
| #11 | 'folacin':ab,ti | 270 |
| #10 | 'folvite':ab,ti | 6 |
| #9 | 'folic acid, (dl)-isomer':ab,ti | 0 |
| #8 | 'folic acid, potassium salt':ab,ti | 0 |
| #7 | 'folic acid, monosodium salt':ab,ti | 0 |
| #6 | 'folic acid, monopotassium salt':ab,ti | 0 |
| #5 | 'pteroylglutamic acid':ab,ti | 407 |
| #4 | 'b9, vitamin':ab,ti | 36 |
| #3 | 'vitamin b9':ab,ti | 368 |
| #2 | 'vitamin m':ab,ti | 16 |
| #1 | 'folic acid'/exp | 77,790 |
| **Cochrane library** | | |
| Search | Query | Results |
| #1 | MeSH descriptor: [Folic Acid] explode all trees | 4,266 |
| #2 | (Vitamin M):ti,ab,kw OR (Vitamin B9):ti,ab,kw OR (B9, Vitamin):ti,ab,kw OR (Pteroylglutamic Acid):ti,ab,kw OR (Folic Acid, Monopotassium Salt):ti,ab,kw | 4,551 |
| #3 | (Folic Acid, Monosodium Salt):ti,ab,kw OR (Folic Acid, Potassium Salt):ti,ab,kw OR (Folvite):ti,ab,kw OR (Folacin):ti,ab,kw AND (Folate):ti,ab,kw | 14 |
| #4 | (diet):ti,ab,kw OR (vegetable):ti,ab,kw AND (fruit):ti,ab,kw | 80,829 |
| #5 | #1 or #2 or #3 or #4 | 88,422 |
| #6 | MeSH descriptor: [Endometrial Neoplasms] explode all trees | 1,042 |
| #7 | (Neoplasm, Endometrial):ti,ab,kw OR (Neoplasms, Endometrial):ti,ab,kw OR (Endometrial Carcinoma):ti,ab,kw OR (Carcinoma, Endometrial):ti,ab,kw OR (Carcinomas, Endometrial):ti,ab,kw | 1,936 |
| #8 | (Endometrial Carcinomas):ti,ab,kw OR (Endometrial Cancer):ti,ab,kw OR (Cancer, Endometrial):ti,ab,kw OR (Cancers, Endometrial):ti,ab,kw OR (Endometrial Cancers):ti,ab,kw | 2,862 |
| #9 | (Endometrium Cancer):ti,ab,kw OR (Cancer, Endometrium):ti,ab,kw OR (Cancers, Endometrium):ti,ab,kw OR (Cancer of the Endometrium):ti,ab,kw OR (Carcinoma of Endometrium):ti,ab,kw | 1,579 |
| #10 | (Endometrium Carcinoma):ti,ab,kw OR (Endometrium Carcinomas):ti,ab,kw OR (Cancer of Endometrium):ti,ab,kw OR (Endometrium Cancers):ti,ab,kw | 1,582 |
| #11 | #6 or #7 or #8 or #9 or #10 | 3,399 |
| #12 | #5 and #11 | 127 |
| **Web of Science** | | |
| Search | Query | Results |
| #1 | TS=( Folic Acid OR Vitamin M OR Vitamin B9 OR B9, Vitamin OR Pteroylglutamic Acid OR Folic Acid, Monopotassium Salt OR Folic Acid, Monosodium Salt OR Folic Acid, Potassium Salt OR Folic Acid, (DL)-Isomer OR Folvite OR Folacin OR Folate OR Folic Acid, (D)-Isomer OR Folic Acid, Calcium Salt (1:1) OR Folic Acid, Sodium Salt OR Diet OR Vegetables OR Fruit) | 1,099,341 |
| #2 | TS=( Endometrial Neoplasms OR Endometrial Neoplasm OR Neoplasm, Endometrial OR Neoplasms, Endometrial OR Endometrial Carcinoma OR Carcinoma, Endometrial OR Carcinomas, Endometrial OR Endometrial Carcinomas OR Endometrial Cancer OR Cancer, Endometrial OR Cancers, Endometrial OR Endometrial Cancers OR Endometrium Cancer OR Cancer, Endometrium OR Cancers, Endometrium OR Cancer of the Endometrium OR Carcinoma of Endometrium OR Endometrium Carcinoma OR Endometrium Carcinomas OR Cancer of Endometrium OR Endometrium Cancers) | 53,444 |
| #3 | #1 AND #2 | 975 |

Supplementary Table 2. Evaluation of the scores of the Newcastle Ottawa Scale for inclusion in the study.
